# Data set characterizing the systemic alterations of microvascular reactivity and capillary density, in patients presenting with infective endocarditis

**DOI:** 10.1016/j.dib.2018.03.039

**Published:** 2018-03-15

**Authors:** Eduardo Tibirica, Amanda Barcelos, Cristiane Lamas

**Affiliations:** a National Institute of Cardiology, Ministry of Health, Rio de Janeiro, Brazil; b National Institute of Infectious Diseases Evandro Chagas, Oswaldo Cruz Institute, Rio de Janeiro, Brazil; c Unigranrio University, Rio de Janeiro, Brazil

## Abstract

This article represents data associated with a prior publication from our research group, under the title: Evaluation of microvascular endothelial function and capillary density in patients with infective endocarditis using laser speckle contrast imaging and video-capillaroscopy [1].

Patients with definite infective endocarditis, under stable clinical conditions, were prospectively included. The clinical and laboratory features are presented for each of them in raw form. Microvascular reactivity was evaluated using a laser speckle contrast imaging (LSCI) system with a laser wavelength of 785 nm. LSCI was used in combination with the iontophoresis of acetylcholine (ACh) or sodium nitroprusside (SNP) for the noninvasive, continuous measurement of cutaneous microvascular perfusion changes in arbitrary perfusion units (APU). The images were analyzed using the manufacturer's software. One skin site on the ventral surface of the forearm was chosen for the experiment. Microvascular reactivity was also evaluated using post-occlusive reactive hyperemia, whereby arterial occlusion was achieved with supra-systolic pressure (50 mmHg above the systolic arterial pressure) using a sphygmomanometer for three minutes. Following the release of pressure, maximum flux was measured. Data on cutaneous microvascular density were obtained using intravital video-capillaroscopy. The data obtained may be helpful by showing the usefulness of laser-based noninvasive techniques in systemic infectious diseases other than sepsis, in different clinical settings and countries.

**Specifications table**

**Table:** 

Subject area	Medicine
More specific subject area	Infectious diseases
Type of data	Tables and figures
How data was acquired	Systemic microvascular reactivity was evaluated using a laser speckle contrast imaging system with (PeriCam PSI system, Perimed, Järfälla, Sweden); Cutaneous capillary density was assessed by high-resolution intra-vital color microscopy (Moritex, Cambridge, UK). Data on patients’ clinical and laboratory features were obtained prospectively from their electronic and written notes
Data format	Raw data on patients’ clinical and laboratory features, analyzed data on microvascular parameters obtained
Experimental factors	The results were presented as the mean±SD. For values that did not follow a Gaussian distribution, the medians (25th - 75th percentiles) were presented (Shapiro- Wilk normality test). The results were analyzed using either two-tailed unpaired Student's t-tests or repeated measures ANOVAs when appropriate
Experimental features	Microvascular reactivity was evaluated using a laser speckle contrast imaging (LSCI) system with a laser wavelength of 785 nm (PeriCam PSI system, Perimed, Järfälla, Sweden). LSCI was used in combination with the iontophoresis of acetylcholine (ACh) or sodium nitroprusside (SNP) for the noninvasive, continuous measurement of cutaneous microvascular perfusion changes in arbitrary perfusion units (APU). The images were analyzed using the manufacturer's software (PIMSoft, Perimed, Järfälla, Sweden). One skin site on the ventral surface of the forearm was chosen for the experiment
Data source location	National Institute of Cardiology, Ministry of Health, Rio de Janeiro, Brazil
Data accessibility	All data are presented in the present article

**Value of the data**

• These data may be valuable to show the usefulness of laser-based noninvasive techniques in systemic infectious diseases in different clinical settings and in various countries.
• These data describe important systemic microvascular features of patients with definite infective endocarditis (IE) and may serve as a benchmark for the detection of microvascular alterations useful in the diagnosis and management of patients with IE.
• These data may be useful for future studies comparing different methodological approaches in the field of study of the microcirculation in infectious diseases.

## 1 Data

These data describe the main features of the systemic microvascular alterations detected in patients presenting with definite infective endocarditis, according to the modified Duke criteria. Both endothelium-dependent and -independent microvascular reactivity were evaluated, using cutaneous micro-iontophoresis of acetylcholine or sodium nitroprusside in the forearm, respectively, associated with laser-based technology. Endothelium-dependent microvascular reactivity was also evaluated using post-occlusive reactive hyperemia in the forearm. The data concerning cutaneous microvascular density were obtained using intravital video-capillaroscopy.

## 2 Experimental design, materials and methods

### 2.1 Study design and place

This is a comparative study that included patients with a confirmed diagnosis of infective endocarditis who were admitted to the National Institute of Cardiology (NIC) at the Ministry of Health in Rio de Janeiro, Brazil. The NIC is a national reference center for the treatment and research of cardiovascular diseases. Its staff is composed of cardiologists, cardiothoracic surgeons, infectious diseases’ specialists, specialized nursing staff, physiotherapists and pharmacists as well as technical staff. The investigative resources include echocardiography, computed tomography, magnetic resonance imaging and scintigraphy. The NIC has outpatient units, four intensive care units and operating theatres where approximately 1300 cardiac surgeries are performed yearly.

### 2.2 Study participants and recruitment

The present study was conducted in accordance with the Declaration of Helsinki 1975, which was revised in 2000, and was approved by the Institutional Review Board (IRB) of the National Institute of Cardiology in Rio de Janeiro, Brazil under protocol # CAAE 52871216.0.0000 and registered at ClinicalTrials.gov (NCT02940340). Study participants were informed on the nature of the protocol and gave written consent.

The eligibility criteria were as follows: i) confirmed IE according to the modified Duke criteria [2]; ii) inpatient treatment at the NIC; iii) clinical stability at the time of the intervention as evaluated by the investigator; iv) age ≥ 18 years and v) cardiac surgery performed more than 15 days prior to the protocol date [3], [4]. The exclusion criterion was a confirmed previous diagnosis of *diabetes mellitus.*

### 2.3 Study variables

The variables included were as follows (Table 1): demographic data (sex and age), medical conditions prior to the diagnosis of IE (systemic arterial hypertension, renal failure on conservative or dialytic treatment, smoking, chronic valvular disease, cardiac surgery or percutaneous procedures), predisposing conditions to IE (previous episode of IE, rheumatic valve disease, congenital heart disease, intravenous drug use, valve prosthesis and intracardiac devices), medications in use (angiotensin-converting enzyme inhibitors (ACEi), angiotensin receptor blockers (ARB), statins, betablockers, diuretics), data referring to the episode of IE, such as timing of presentation (acute IE was defined as the presentation of signs and symptoms within one month of diagnosis, and subacute IE as that presenting for more than one month at the time of diagnosis), mode of acquisition (community-acquired and health care-related; the second defined as IE occurring more than 72 hours following hospital admission or acquired within two months of an invasive procedure), etiologic agents. These latter were divided into four groups for analysis: i) viridans group streptococci, including those with blood culture negative, since we have previously shown by PCR of excised valves that viridians streps are the most frequent agents in our scenario IE [5]; ii) aggressive staphylococci (including *Staphylococcus aureus* both methicillin susceptible and resistant and *S. lugdunensis*; iii) coagulase negative staphylococci and iv) enterococci. We also evaluated affected structures and left ventricular function, evaluated as normal or moderately to severely compromised at the time of diagnosis of IE; left ventricular ejection fraction was not used as a parameter for heart dysfunction as it is often overestimated in moderate to severe valvular regurgitation, the predominant lesion in IE. Other variables studied were pulmonary artery systolic pressure (PASP), as an indirect measure of myocardial dysfunction, embolic and non-embolic complications (paravalvular abscess, prosthetic dehiscence, atrioventricular block, new cardiac failure, new renal failure); antibiotic and surgical treatment and laboratory data (C-reactive protein levels, CRP, hemoglobin, hematocrit, leukocyte count, and serum creatinine levels).

**Table 1:** Clinical and laboratory features of 22 patients with definite infective endocarditis.

**Patients with infective endocarditis**	Sex	Age	Affected valve	Place of acquisition	Causative pathogen	Previous IE	Rheumatic valvopathy	CHD
Patient 1	M	70	Aortic	Community	*Staphylococcus.epidermidis*	No	No	No
Patient 2	F	30	Mitral	Community	*Viridans* group streptococcus	No	No	No
Patient 3	M	40	Mitral	Community	*Granulicatella*	No	No	No
Patient 4	M	46	Aortic	Community	*Viridans* group streptococcus	Yes	No	Yes
Patient 5	F	68	Mitral	Community	*S. lugdunensis*	No	No	No
Patient 6	F	65	Mitral	Hospital	*Granulicatella*	No	No	No
Patient 7	M	33	Aortic	Community	*Viridans* group streptococcus	No	No	Yes
Patient 8	M	44	Mitral	Community	*Bovis* group streptococcus	No	No	No
Patient 9	F	37	Mitral	Community	*Enterococcus faecalis*	No	No	Yes
Patient 10	M	55	Mitral	Community	*S. epidermidis*	No	No	No
Patient 11	F	67	Aortic	Community	*E. faecalis*	No	No	No
Patient 12	M	38	Aortic	Community	*E. faecalis*	No	No	No
Patient 13	F	42	Aortic	Community	*E. faecalis*	No	No	No
Patient 14	M	66	Mitral	Community	BCNE	No	No	No
Patient 15	M	26	Mitral	Community	BCNE	No	No	No
Patient 16	M	22	Mitral	Hospital	MSSA	No	Yes	Yes
Patient 17	M	62	Aortic	Community	*S.epidermidis*	No	No	No
Patient 18	F	41	Mitral	Community	*Viridans* group streptococcus	Yes	Yes	No
Patient 19	F	24	Tricuspid	Hospital	MSSA	No	No	Yes
Patient 20	M	72	Aortic	Community	*S.epidermidis*	No	No	No
Patient 21	M	18	Aortic	Community	MSSA	No	No	Yes
Patient 22	M	35	Mitral	Community	BCNE	No	No	No


**Patients with infective endocarditis**	IVDU	Valve prosthesis	Pacemaker	CRF	HD	Hypertension	Dyslipidemia	LV systolic dysfunction complicating IE
Patient 1	No	No	Yes	No	No	Yes	Yes	No
Patient 2	No	No	No	No	No	No	No	No
Patient 3	No	No	No	No	No	No	No	No
Patient 4	No	No	No	No	No	No	No	No
Patient 5	No	Yes	Yes	No	No	Yes	No	No
Patient 6	No	No	Yes	No	No	Yes	No	No
Patient 7	No	No	Yes	No	No	Yes	No	No
Patient 8	No	No	No	No	No	No	No	Yes
Patient 9	No	No	No	No	No	No	No	No
Patient 10	No	No	Yes	Yes	No	No	No	No
Patient 11	No	No	No	No	No	Yes	No	No
Patient 12	No	No	Yes	No	No	No	No	No
Patient 13	Yes	No	Yes	No	No	Yes	No	No
Patient 14	No	No	Yes	Yes	Yes	Yes	Yes	No
Patient 15	No	No	No	No	No	Yes	No	No
Patient 16	No	No	Yes	No	No	No	No	No
Patient 17	No	No	Yes	No	No	Yes	No	Yes
Patient 18	No	No	No	Yes	No	Yes	Yes	No
Patient 19	No	No	Yes	No	No	No	No	No
Patient 20	No	Yes	Yes	No	Yes	Yes	No	No
Patient 21	No	No	No	Yes	Yes	Yes	No	No
Patient 22	Yes	No	No	No	No	No	No	Yes

IE, Infective endocarditis; M, Male; F, Female; MSSA, methicillin susceptible *Staphylococcus aureus*; BCNE, blood culture negative; CHD, Congenital heart disease; IVDU; Intravenous drug user; LV, Left ventricle; CRF, chronic renal failure; HD, hemodialysis.								


**Patients with infective endocarditis**	Hematocrit (%)	Creatinine (mg/dL)	C reactive protein (mg/dL)
Patient 1	36.8	0.59	0.65
Patient 2	40.3	0.94	0.48
Patient 3	35.2	1.27	1.92
Patient 4	40.4	0.95	0.24
Patient 5	29.2	1.16	0.74
Patient 6	33.6	0.85	2.89
Patient 7	37.2	0.87	2.56
Patient 8	37.2	0.72	3.72
Patient 9	25.2	1.5	3.72
Patient 10	21.8	0.99	0.75
Patient 11	29.8	0.93	2.12
Patient 12	34.2	1.02	0.35
Patient 13	36.4	2.9	1.17
Patient 14	28.1	0.94	0.93
Patient 15	32.1	0.82	5.75
Patient 16	38.4	0.78	0.24
Patient 17	39.4	1.79	0.52
Patient 18	28.0	0.93	7.75
Patient 19	26.0	3.31	17.34
Patient 20	29.0	0.98	5.66
Patient 21	28.0	1.02	4.47
Patient 22	41.0	1.43	7.68

### 2.4 Intervention

The evaluation of microvascular endothelial function in patients with infective endocarditis was performed using laser speckle contrast imaging. These results were compared to those previously obtained from age and sex-matched healthy volunteers [6]. The systemic microvascular data obtained from this group of healthy volunteers were used as reference microcirculatory values of individuals free of systemic diseases. The healthy volunteers did not present with arterial hypertension, diabetes, dyslipidemia or any other systemic pathology.

### 2.5 Evaluation of microcirculatory reactivity

The microcirculatory tests were performed in the morning between 8 A.M. and 12 P.M. in an undisturbed, quiet room with a defined stable temperature (23 ± 1 °C), following a 20-min rest period in the supine position. The room temperature was monitored and adjusted if necessary using air conditioning. The acclimatization period lasted until the patient's skin temperature stabilized [7]. We have previously demonstrated that following 15–20 min of acclimatization, the skin temperature stabilizes at approximately 29 °C [8].

### 2.6 Evaluation of skin microvascular flow and reactivity

**Fig. 1:**
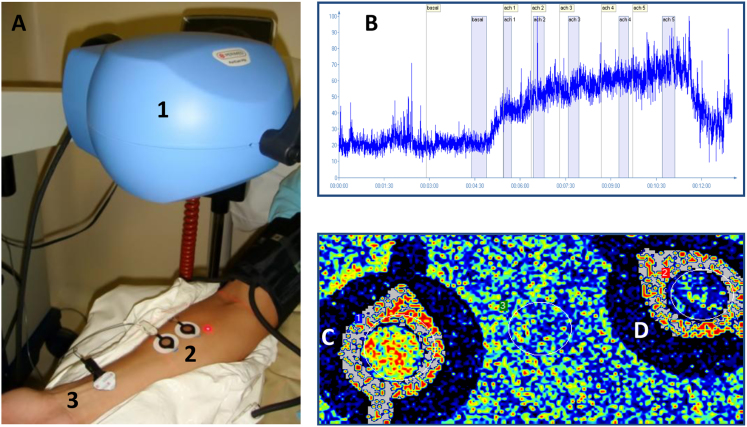
Picture of the experimental set up used in the assessment of skin microvascular perfusion using laser speckle contrast imaging coupled with iontophoresis of vasodilator drugs (A). Representative example of the effects of the transdermal iontophoretic delivery of cumulative doses of acetylcholine on skin blood flow (B) and video images of the acetylcholine iontophoresis (C) compared to a vehicle-containing electrode (D). (1) imager head, (2) drug-delivery iontophoresis electrodes, (3) dispersive electrode (see methods).

Microvascular reactivity was evaluated using a laser speckle contrast imaging (LSCI) system with a laser wavelength of 785 nm (PeriCam PSI system, Perimed, Järfälla, Sweden), as previously described [9] (Fig. 1). LSCI was used in combination with the iontophoresis of acetylcholine (ACh) or sodium nitroprusside (SNP) for the noninvasive, continuous measurement of cutaneous microvascular perfusion changes in arbitrary perfusion units (APU) (Table 2, Table 3, Table 4). The images were analyzed using the manufacturer's software (PIMSoft, Perimed, Järfälla, Sweden). One skin site on the ventral surface of the forearm was randomly chosen for the recordings. Hair, broken skin, areas of skin pigmentation and visible veins were avoided, and a single drug-delivery electrode was installed using adhesive discs (LI 611, Perimed, Järfälla, Sweden). A vacuum cushion (AB Germa, Kristianstad, Sweden) was used to reduce the recording artifacts generated by arm movements. The iontophoresis of ACh 2% w/v or SNP 2% w/v (Sigma Chemical CO, USA) was performed using a micropharmacology system (PF 751 PeriIont USB Power Supply, Perimed, Sweden) with increasing anodal currents of 30, 60, 90, 120, 150 and 180 μA for 10-second intervals that are spaced 1 min apart, and the total charges were 0.3, 0.6, 0.9, 1.2, 1.5 and 1.8 mC. The dispersive electrode was attached approximately 15 cm away from the iontophoresis chamber. The results of the pharmacological tests were expressed both as peak values (representing the maximal vasodilation observed following the highest dose of ACh or SNP) and as the area under the curve of vasodilation. The measurements of skin blood flow were divided by mean arterial pressure values to provide the cutaneous vascular conductance (CVC) in APUs/mmHg. Microvascular reactivity was also evaluated using a physiological test known as post-occlusive reactive hyperemia (PORH). During the PORH test, arterial occlusion was achieved with supra-systolic pressure (50 mmHg above the systolic arterial pressure) using a sphygmomanometer for three minutes. Following the release of pressure, maximum flux was measured. PORH was not performed in four patients due to technical reasons.

**Table 2:** Values of systemic microvascular flow expressed as cutaneous microvascular conductance (CVC), calculated as arbitrary perfusion units (APU) divided by mean arterial pressure, in mmHg, obtained after skin iontophoresis of acetylcholine or sodium nitroprusside in patients presenting with infective endocarditis (n = 22) or age- and sex-matched healthy individuals (n = 30).

**Current intensity of acetylcholine iontophoresis (µA)**	**Patients with infective endocarditis**						
	0	30	60	90	120	150	180
CVC (APU/mmHg)							
Mean	0.35	0.41	0.42	0.43	0.46*	0.51**	0.56***
SD	0.13	0.18	0.21	0.18	0.20	0.22	0.24
SEM	0.03	0.04	0.05	0.04	0.04	0.05	0.05
Median	0.35	0.39	0.37	0.38	0.40	0.45	0.49
25% Percentile	0.23	0.26	0.27	0.30	0.34	0.37	0.41
75% Percentile	0.49	0.51	0.55	0.57	0.61	0.67	0.74
							
**Current intensity of sodium nitroprusside iontophoresis (µA)**	0	30	60	90	120	150	180
CVC (APU/mmHg)							
Mean	0.36	0.35	0.37	0.38	0.42	0.46	0.53
SD	0.13	0.12	0.14	0.12	0.15*	0.18**	0.21***
SEM	0.03	0.03	0.03	0.03	0.03	0.04	0.05
Median	0.30	0.29	0.33	0.36	0.37	0.39	0.47
25% Percentile	0.27	0.26	0.26	0.28	0.31	0.33	0.37
75% Percentile	0.47	0.45	0.45	0.46	0.55	0.58	0.66
	
	**Reference values from healthy individuals**						
**Current Intensity of Acetylcholine Iontophoresis (µA)**	0	30	60	90	120	150	180
CVC (APU/mmHg)							
Mean	0.21	0.25	0.32	0.40*	0.46*	0.51**	0.58***
SD	0.08	0.10	0.12	0.15	0.17	0.18	0.18
SEM	0.01	0.02	0.02	0.03	0.03	0.04	0.04
Median	0.20	0.23	0.32	0.42	0.48	0.50	0.58
25% Percentile	0.15	0.18	0.22	0.25	0.28	0.34	0.44
75% Percentile	0.27	0.29	0.41	0.49	0.59	0.65	0.72
							
**Current Intensity of Sodium Nitroprusside Iontophoresis (µA)**	0	30	60	90	120	150	180
CVC (APU/mmHg)							
Mean	0.21	0.22	0.23**	0.28****	0.34****	0.40****	0.50****
SD	0.07	0.08	0.07	0.09	0.13	0.16	0.20
SEM	0.01	0.01	0.01	0.03	0.03	0.03	0.04
Median	0.20	0.20	0.23	0.27	0.33	0.38	0.46
25% Percentile	0.15	0.15	0.17	0.21	0.24	0.29	0.38
75% Percentile	0.27	0.26	0.31	0.34	0.45	0.49	0.62

APU, arbitrary perfusion units; CVC, Cutaneous microvascular conductance; SD, standard deviation; SEM, standard error of the mean.

* P < 0.05 compared to basal values.

** P < 0.01 compared to basal values.

*** P < 0.001 compared to basal values.

**** P < 0.0001 compared to basal values.

**Table 3:** Values of systemic microvascular flow expressed as cutaneous microvascular conductance (CVC), calculated as arbitrary perfusion units (APU) divided by mean arterial pressure, in mmHg, obtained before and after skin iontophoresis of acetylcholine or sodium nitroprusside in patients presenting with infective endocarditis (n = 22) or age- and sex-matched healthy individuals (n = 30).

	**Patients with infective endocarditis**	**Reference values from healthy individuals**
**Baseline values**		
CVC (APU/mmHg)		
Mean	0.36****	0.21
SD	0.13	0.08
SEM	0.03	0.01
Median	0.35	0.20
25% Percentile	0.23	0.15
75% Percentile	0.49	0.28
		
**Peak values after acetylcholine iontophoresis**		
CVC (APU/mmHg)		
Mean	0.56	0.58
SD	0.24	0.18
SEM	0.05	0.04
Median	0.49	0.58
25% Percentile	0.40	0.44
75% Percentile	0.74	0.72
		
**Peak values after sodium nitroprusside iontophoresis**		
CVC (APU/mmHg)		
Mean	0.53	0.50
SD	0.21	0.20
SEM	0.04	0.04
Median	0.47	0.45
25% Percentile	0.37	0.39
75% Percentile	0.66	0.61
		
**Increase in CVC after acetylcholine iontophoresis (peak – baseline)**		
CVC (APU/mmHg)		
Mean	0.21**	0.37
SD	0.17	0.14
SEM	0.04	0.03
Median	0.19	0.39
25% Percentile	0.11	0.26
75% Percentile	0.26	0.5
		
**Increase in CVC after sodium nitroprusside iontophoresis (peak – baseline)**		
CVC (APU/mmHg)		
Mean	0.18*	0.29
SD	0.14	0.15
SEM	0.03	0.03
Median	0.14	0.26
25% Percentile	0.10	0.23
75% Percentile	0.25	0.34
		
**Area under the curve of acetylcholine iontophoresis**		
APU/s		
Mean	20,092	18,539
SD	6850	6033
SEM	1495	1207
Median	19,262	19,674
25% Percentile	15,935	11,567
75% Percentile	25,199	23,651
		
**Area under the curve of sodium nitroprusside iontophoresis**		
APU/s		
Mean	17,975	15,117
SD	4928	4972
SEM	1102	994
Median	26,663	14,628
25% Percentile	14,396	11,152
75% Percentile	21,000	20,733

APU, arbitrary perfusion units; CVC, Cutaneous microvascular conductance; SD, standard deviation; SEM, standard error of the mean.

* P < 0.05 compared to healthy individuals.

** P < 0.01 compared to healthy individuals.

**** P < 0.0001 compared to healthy individuals.

**Table 4:** Values of systemic microvascular flow expressed as cutaneous microvascular conductance (CVC), calculated as arbitrary perfusion units (APU) divided by mean arterial pressure, in mmHg, obtained after post-occlusive reactive hyperemia in patients presenting with infective endocarditis (n = 10) or age- and sex-matched healthy individuals (n = 25).

	**Patients with infective endocarditis**	**Reference values from healthy individuals**
**Baseline values**		
CVC (APU/mmHg)		
Mean	0.36****	0.21
SD	0.13	0.08
SEM	0.03	0.01
Median	0.35	0.20
25% Percentile	0.23	0.15
75% Percentile	0.49	0.28
		
**Peak values after PORH**		
CVC (APU/mmHg)		
Mean	0.82	0.74
SD	0.28	0.19
SEM	0.09	0.04
Median	0.80	0.74
25% Percentile	0.56	0.59
75% Percentile	1.01	0.85
		
**Increase in CVC after PORH (peak – baseline)**		
CVC (APU/mmHg)		
Mean	0.45	0.46
SD	0.20	0.17
SEM	0.06	0.03
Median	0.40	0.47
25% Percentile	0.31	0.33
75% Percentile	0.63	0.55

APU, arbitrary perfusion units; CVC, Cutaneous microvascular conductance; PORH, post-occlusive reactive hyperemia; SD, standard deviation; SEM, standard error of the mean.

**** P < 0.0001 compared to healthy individuals.

### 2.7 Capillaroscopy by intravital video-microscopy

**Fig. 2:**
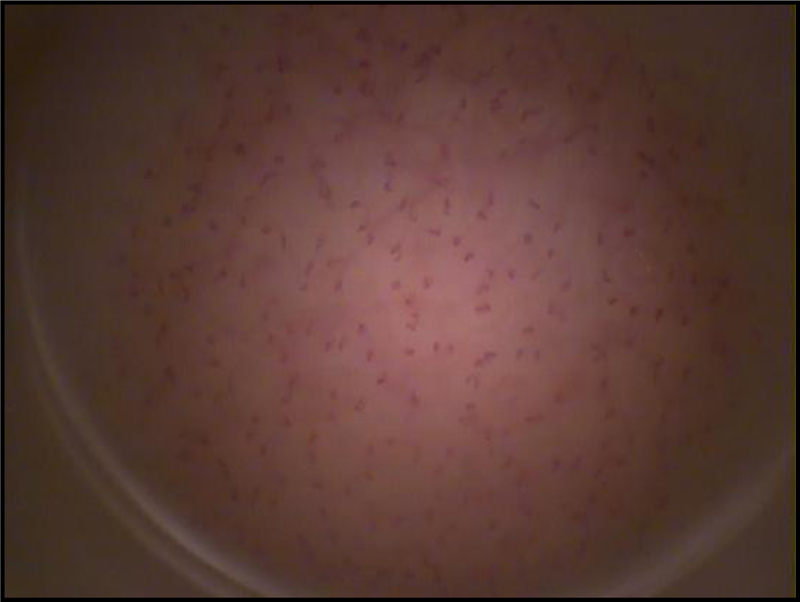
Representative image of perfused capillaries of the skin of the finger, from a healthy control subject, obtained with high-resolution intra-vital color video microscopy and shown at a final magnification of 200×. For each patient, the mean capillary density was calculated as the arithmetic mean of the number of spontaneously perfused capillaries in three contiguous microscopic fields of 1 mm^2^ each.

The dorsum of the non-dominant middle phalanx was used for image acquisition, while keeping the patient sitting comfortably. The arm was positioned at the level of the heart and immobilized using a vacuum cushion (a specially constructed pillow filled with polyurethane foam that can be molded to any desired shape by creating a vacuum, from AB Germa, Kristianstad, Sweden). Capillary density, i.e., the number of spontaneously perfused capillary loops per square millimeter of skin area, was assessed by high-resolution intra-vital color microscopy (Moritex, Cambridge, UK), as previously described and validated [8], [10], [11]. We used a video-microscopy system with an epi-illuminated fiber optic microscope containing a 100-W mercury vapor lamp light source and an M200 objective with a final magnification of 200×. Images were acquired and saved for posterior off-line analysis using a semi-automatic integrated system (Saisam, Microvision Instruments, Evry, France). The mean capillary density for each patient was calculated as the arithmetic mean of visible (i.e., spontaneously perfused) capillaries in three contiguous microscopic fields of 1 mm^2^ each (Fig. 2 and Table 5). The mean number of skin spontaneously perfused capillaries at rest is considered to represent the functional capillary density, as previously described [12]. The features of the study subjects were not available to the investigator during capillary counting. Reproducibility was assessed by examining an identical area of skin. Intra-observer repeatability of data analysis was assessed by reading the same images blindly on two separate occasions (n = 15, coefficient of variability 4.3%).

**Table 5:** Values of skin capillary density, obtained using high-resolution intra-vital color video-microscopy, in patients presenting with infective endocarditis (n = 9) or age- and sex-matched healthy individuals (n = 30).

**Capillary density (capillaries/mm**^**2**^**)**	**Patients with infective endocarditis**	**Reference values from healthy individuals**
Mean	135****	97
SD	24	21
SEM	8	4
Median	143	96
25% Percentile	115	80
75% Percentile	148	110

SD, standard deviation; SEM, standard error of the mean.

**** P < 0.0001 compared to healthy individuals.

### 2.8 Statistical analysis

The results were presented as the mean±SD. For values that did not follow a Gaussian distribution, the medians (25th–75th percentiles) were presented (Shapiro- Wilk normality test). The results were analyzed using either two-tailed unpaired Student's *t-*tests or repeated measures ANOVAs when appropriate. The independent (unpaired) t-test was used because we compared two unrelated groups, in which the participants (healthy individuals or patients with IE) in each group are different. P values < 0.05 were considered statistically significant. Clinical and laboratory data were shown descriptively. The correlations between the intervention study results (microvascular reactivity) and features of the disease, such as the number of days of presentation, presence of embolic complications and etiological agent, were determined using Pearson's test if the data are found to be of normal distribution (parametric). If the distribution was not normal (non-parametric), Spearman's test was used for the analysis. The identification of potential outliers was performed using the ROUT method (robust regression and outlier removal), which is based on the False Discovery Rate (FDR), with a specified value of Q = 1%.The statistical package used for the statistical analyses was Prism version 6.0 (GraphPad Software Inc. La Jolla, CA, USA) and the R version 3.1.0.

## References

[1] Barcelos A., Tibirica E., Lamas C. (2018). Evaluation of microvascular endothelial function and capillary density in patients with infective endocarditis using laser speckle contrast imaging and video-capillaroscopy. Microvasc. Res..

[2] Li J.S., Sexton D.J., Mick N., Nettles R., Fowler V.G., Ryan T., Bashore T., Corey G.R. (2000). Proposed modifications to the Duke criteria for the diagnosis of infective endocarditis. Clin. Infect. Dis..

[3] Wan S., LeClerc J.L., Vincent J.L. (1997). Cytokine responses to cardiopulmonary bypass: lessons learned from cardiac transplantation. Ann. Thorac. Surg..

[4] Boralessa H., de Beer F.C., Manchie A., Whitwam J.G., Pepys M.B. (1986). C-reactive protein in patients undergoing cardiac surgery. Anaesthesia.

[5] Lamas C.C., Fournier P.E., Zappa M., Brandao T.J., Januario-da-Silva C.A., Correia M.G., Barbosa G.I., Golebiovski W.F., Weksler C., Lepidi H., Raoult D. (2016). Diagnosis of blood culture-negative endocarditis and clinical comparison between blood culture-negative and blood culture-positive cases. Infection.

[6] Souza E.G., De Lorenzo A., Huguenin G., Oliveira G.M., Tibirica E. (2014). Impairment of systemic microvascular endothelial and smooth muscle function in individuals with early-onset coronary artery disease: studies with laser speckle contrast imaging. Coron. Artery Dis..

[7] Shore A.C. (2000). Capillaroscopy and the measurement of capillary pressure. Br. J Clin. Pharmacol..

[8] Tibirica E., Rodrigues E., Cobas R.A., Gomes M.B. (2007). Endothelial function in patients with type 1 diabetes evaluated by skin capillary recruitment. Microvasc. Res..

[9] Cordovil I., Huguenin G., Rosa G., Bello A., Kohler O., de Moraes R., Tibirica E. (2012). Evaluation of systemic microvascular endothelial function using laser speckle contrast imaging. Microvasc. Res..

[10] Kaiser S.E., Sanjuliani A.F., Estato V., Gomes M.B., Tibirica E. (2013). Antihypertensive treatment improves microvascular rarefaction and reactivity in low-risk hypertensive individuals. Microcirculation.

[11] Francischetti E.A., Tibirica E., da Silva E.G., Rodrigues E., Celoria B.M., de Abreu V.G. (2011). Skin capillary density and microvascular reactivity in obese subjects with and without metabolic syndrome. Microvasc. Res..

[12] Tibirica E., Souza E.G., De Lorenzo A., Oliveira G.M. (2015). Reduced systemic microvascular density and reactivity in individuals with early onset coronary artery disease. Microvasc. Res..

