# Supplementary material for: Data set characterizing the systemic alterations of microvascular reactivity and capillary density, in patients presenting with infective endocarditis
Source: Data Brief. 2018 Mar 15;18:480–91. doi: 10.1016/j.dib.2018.03.039 (PMC5996261; doi:10.1016/j.dib.2018.03.039)
Supplement: Supplementary file 1 — Supplementary material [file mmc1.docx]

**DISCLOSURES**

The authors have no conflicts to disclose.
